# Relationship between systemic immune-inflammation index and osteoarthritis: a cross-sectional study from the NHANES 2005–2018

**DOI:** 10.3389/fmed.2024.1433846

**Published:** 2024-08-14

**Authors:** Qiang He, Zhen Wang, Jie Mei, Chengxin Xie, Xin Sun

**Affiliations:** ^1^Nanjing Hospital of Chinese Medicine Affiliated to Nanjing University of Chinese Medicine, Nanjing, China; ^2^Affiliated Hospital of Shandong University of Chinese Medicine, Jinan, China; ^3^QiQiHaEr Traditional Chinese Medicine Hospital, QiQiHaEr, China; ^4^Shandong First Medical University, Jinan, China; ^5^Department of Orthopedics, Taizhou Hospital of Zhejiang Province Affiliated to Wenzhou Medical University, Taizhou, China

**Keywords:** systemic immune-inflammation index, osteoarthritis, NHANES, relationship, a cross-sectional study

## Abstract

**Objective:**

The study aimed to explore the relationship between systemic inflammatory response index (SIRI) levels and osteoarthritis (OA) using cross-sectional data from the National Health and Nutrition Examination Survey (NHANES) database from 2005 to 2018.

**Methods:**

Using cross-sectional data from the NHANES database from 2005 to 2018, we included 11,381 study participants divided into OA (*n* = 1,437) and non-OA (*n* = 9,944) groups. Weighted multivariable regression models and subgroup analyses were employed to investigate the relationship between SIRI and OA. Additionally, restricted cubic spline models were used to explore nonlinear relationships.

**Results:**

This study enrolled 11,381 participants aged ≥20 years, including 1,437 (14%) with OA. Weighted multivariable regression analysis in the fully adjusted Model 3 indicated a correlation between higher levels of SIRI (log_2_-transformed) and an increased OA risk (odds ratio: 1.150; 95% confidence interval: 1.000–1.323, *p* < 0.05). Interaction tests showed that the variables did not significantly affect this correlation (*p* for interaction all >0.05). Additionally, a restricted cubic spline model revealed a nonlinear relationship between log_2_(SIRI) and OA risk, with a threshold effect showing 4.757 as the critical value of SIRI. SIRI <4.757 showed almost unchanged OA risk, whereas SIRI >4.757 showed rapidly increasing OA risk.

**Conclusion:**

The positive correlation between SIRI and OA risk, with a critical value of 4.757, holds clinical value in practical applications. Additionally, our study indicates that SIRI is a novel, clinically valuable, and convenient inflammatory biomarker that can be used to predict OA risk in adults.

## 1 Introduction

Osteoarthritis (OA) is a common chronic degenerative joint disease characterized by joint dysfunction, pain, and stiffness as its main clinical manifestations (1). A study published in The Lancet on the global trends and future projections of OA indicated that by 2020, OA had become the 15th leading cause of disability worldwide, affecting over 500 million people globally (2). Currently, the primary management of early to mid-stage OA involves pharmacotherapy, whereas joint replacement surgery remains the only effective treatment for end-stage disease, although the implants have a limited lifespan (3, 4). Consequently, the prevention and control of OA should focus on early detection, diagnosis, and treatment, as well as on reducing modifiable risk factors among the population.

The etiology of OA involves multiple factors, including age, genetic susceptibility, obesity, and inflammation, among which inflammation represents a significant risk factor (5, 6). The cellular microenvironment associated with OA is exceedingly complex, involving various cell types and a series of cytokines they secrete (7). Cytokines establish intricate inflammatory networks through endocrine, autocrine, and paracrine stimulation of tissues. Previous studies (8, 9) have indicated that changes in peripheral blood leukocytes, lymphocytes, neutrophils, platelets, red cell distribution width, and certain acute phase protein levels can reflect changes in the body’s inflammatory response. Therefore, various peripheral blood parameters combination models, such as the neutrophil-to-lymphocyte ratio (NLR), monocyte-to-lymphocyte ratio (MLR), and prognostic nutritional index, have been suggested to be correlated with OA (10). The systemic inflammatory response index (SIRI), which is based on the ratios of neutrophils, monocytes, and lymphocytes, comprehensively reflects the body’s inflammatory and immune balance state.

Recently, SIRI has been used as a predictive indicator of mortality in patients with cancer (11–13). Chao et al. (12) found that a high SIRI level is associated with advanced clinical stages and poor prognosis in cervical cancer. SIRI is a more accurate predictor of prognosis in patients with cervical cancer than the NLR, platelet-to-lymphocyte ratio, and MLR. Additionally, SIRI is significantly correlated with TNM stage, endocrine therapy, and overall survival in patients with breast cancer and can independently predict overall survival. The systemic immune-inflammation index (SII), which combines platelet, neutrophil, and lymphocyte counts, effectively reflects tumor prognosis and inflammatory immune status. Cheng et al. (13) found that in patients with asthma, higher levels of SII and SIRI were associated with significantly increased stroke prevalence, with a stronger association observed in individuals with obesity and hyperlipidemia comorbidities. SII and SIRI are relatively stable novel inflammatory markers in patients with asthma, but SIRI provides a better predictive value for stroke prevalence than SII. However, to date, no studies have explored the correlation between SIRI levels and OA risk. Therefore, this study aimed to explore the relationship between SIRI levels and the risk of OA. We hypothesized that patients with OA will have higher SIRI levels.

## 2 Materials and methods

### 2.1 Study design and population

The National Health and Nutrition Examination Survey (NHANES) is a principal project of the National Center for Health Statistics (NCHS), designed as a complex, multi-stage probability sample to assess the health and nutritional status of adults and children in the United States (14). All adult participants in the study signed an informed written consent form. The study was approved by the Institutional Review Board of the NCHS. Venous blood was drawn from participants, and serum samples were processed, stored, and then transported to a collaborative laboratory service department for analysis. The details of the biological samples that were collected can be obtained on the website.^1^

**Figure 1:**
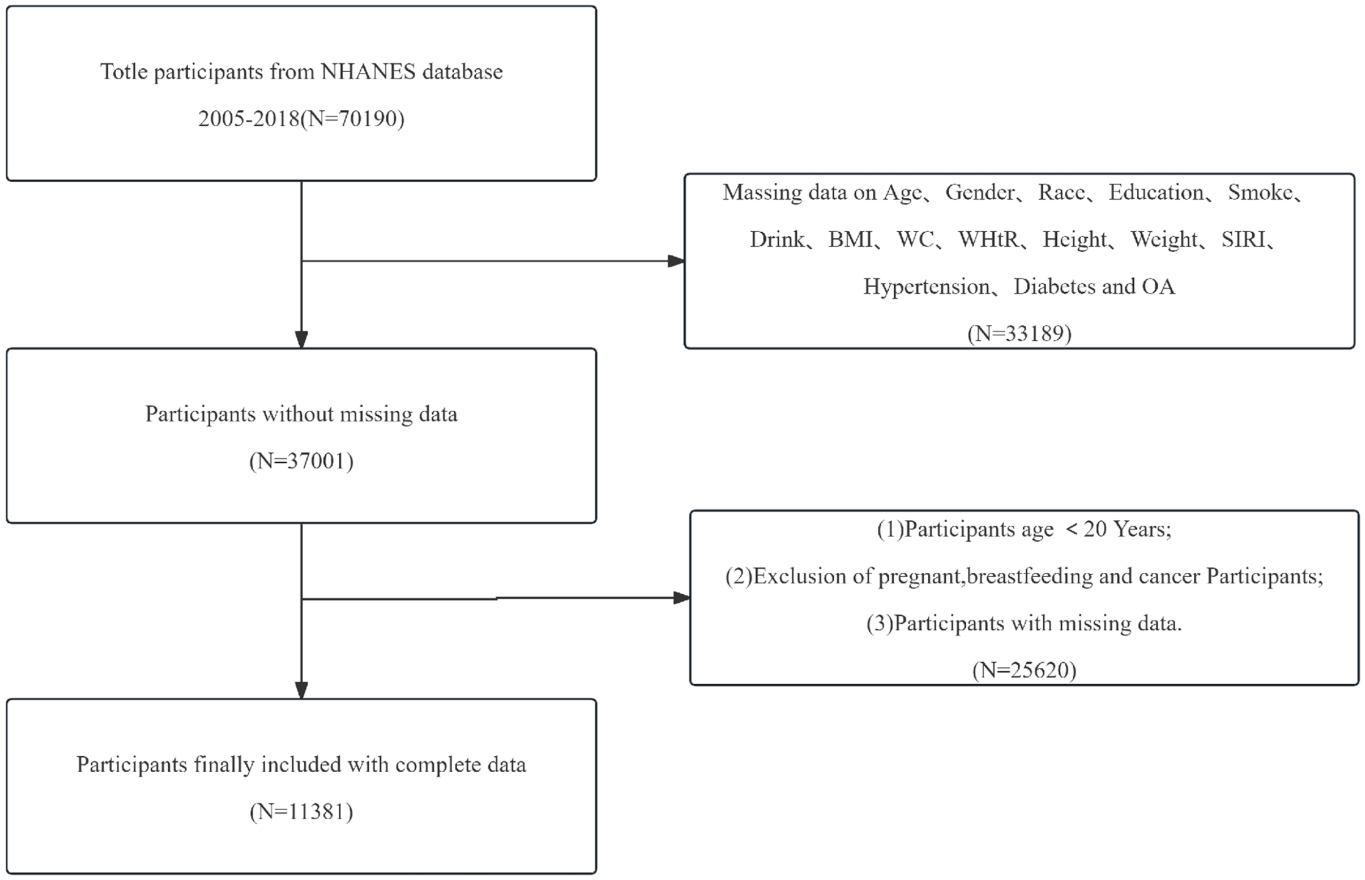
Flow chart of participants selection.

This study utilized cross-sectional data from 70,190 participants in the NHANES database from 2005 to 2018. After excluding 58,809 individuals aged <20 years, those who were pregnant or breastfeeding, patients with cancer, and those lacking other covariate data, a total of 11,381 participants were included in the final analysis. Figure 1 illustrates the screening process.

### 2.2 Definitions of waist-to-height ratio, visceral adiposity index, and OA

#### 2.2.1 Body mass index

According to the guidelines proposed by the World Health Organization (WHO) in 2008 (15), BMI classifications are as follows: normal group: 18.5 kg/m^2^ ≤ BMI <25 kg/m^2^; overweight group: 25 kg/m^2^ ≤ BMI < 30 kg/m^2^; and obesity group: ≥30 kg/m^2^.

#### 2.2.2 Waist circumference

According to the 26th European Congress on Obesity (2019 CEO) (16), a WC of ≥85 cm in males and ≥ 80 cm in females is considered above the standard, classifying them in the WC excess group.

#### 2.2.3 Waist-to-height ratio

In this study, the WHtR was calculated as WC (cm) divided by height (cm). The 26th European Congress on Obesity (2019 CEO) (16) defines a WHtR of ≥0.5 (50%) as abdominal obesity, classifying it as the WHtR excess group.

#### 2.2.4 Systemic inflammatory response index

**Figure 2:**
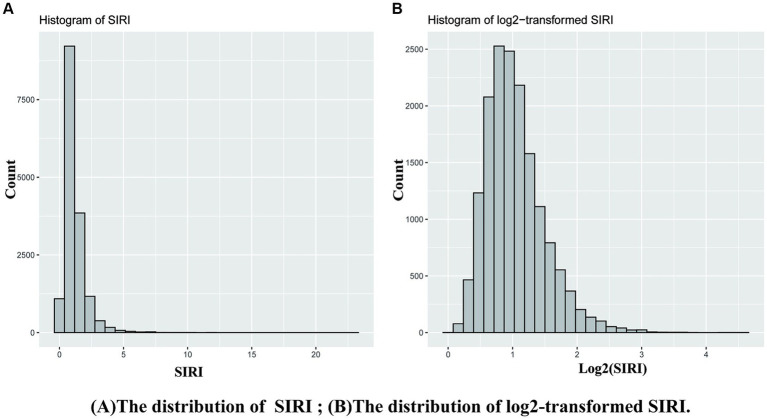
The logarithmic transformation of SIRI. **(A)** SIRI **(B)** Log2(SIRI).

The formula used in this study for calculating the SIRI (13) was (neutrophil count × monocyte count)/lymphocyte count. SIRI was found not to follow a normal distribution (Figure 2A); therefore, we applied a log_2_ transformation to SIRI for regression analysis (Figure 2B).

#### 2.2.5 Systemic immune-inflammation index

The SII was calculated using the following formula: SII = platelet count × (neutrophil count/lymphocyte count). The platelet, neutrophil, and lymphocyte counts were measured in 1,000 cells/mL.

#### 2.2.6 Osteoarthritis

In this study, the inclusion of participants as OA and non-OA groups was based on self-reported OA status from a questionnaire survey (17). This involved two parts: (1) Participants were asked, “Has a doctor or other health professional ever told you that you have arthritis?” Those who answered “yes” were considered for the next question; (2) They were then asked, “What type of arthritis is this?” Participants who answered “OA” were classified as having OA.

### 2.3 Covariates

The covariates included in this study were selected based on their correlation with OA and inclusion in similar past research, encompassing a range of demographic and health-related variables (17). Specifically, we included age (categorized according to the WHO’s age division of adult and older adult groups: 20–59 years and ≥ 60 years, respectively) (18), sex (categorized as male and female), race (categorized as Mexican-American, non-Hispanic White, non-Hispanic Black, other Hispanic, and other race), education level (categorized as below high school, high school, and above high school), smoking status (categorized as never smoker, former smoker, and current smoker), alcohol consumption (categorized as <12 drinks/year and ≥ 12 drinks/year), hypertension (categorized as hypertensive and non-hypertensive), and diabetes mellitus (DM) (categorized as DM and non-DM). Detailed measurement techniques for the research variables are available at www.cdc.gov/nchs/nhanes/, the official website of the Centers for Disease Control and Prevention.

### 2.4 Statistical analysis

This study utilized the NHANES database. Given the complexity of NHANES’ sampling design, the analysis and reporting guidelines were followed to weigh the participant samples from 2005 to 2018, ensuring national representativeness. Data analysis was performed using R software version 4.2.1 and Empower Stats (version 2.0). The geometric means of SIRI and OA and their 95% confidence intervals (CI) were presented, with continuous variables expressed as geometric means and their CIs and categorical variables as weighted frequency distributions. Continuous variables were analyzed using survey-weighted linear regression, and categorical variables using survey-weighted chi-square tests to compare baseline characteristics.

Before analysis, we found that SIRI did not follow a normal distribution; therefore, we applied log_2_ transformation to SIRI. The relationship between log_2_(SIRI) and the risk of OA was analyzed using univariate and weighted multivariable logistic regression. Initially, a crude model (Model 1) was analyzed. Subsequently, adjustments were made for age, sex, and race (Model 2), and finally, for age, sex, race, educational level, BMI, WHtR, smoking, drinking, hypertension, and DM (Model 3). Additionally, log_2_(SIRI) was modeled as a continuous variable based on quartiles [Quartile 1 (Q1): 25th percentile, Quartile 2: 25–50th percentile, Quartile 3: 50–75th percentile, and Quartile 4 (Q4): 75–100th percentile]. Three multivariable logistic regression models (as above) were used to estimate the odds ratios (ORs) and 95% CIs of the association between log_2_(SIRI) and OA. A linear trend (dose–response relationship) was estimated by specifying the median log_2_(SIRI) value of each quartile as a continuous variable, and a restricted cubic spline model was used to explore the nonlinear association between log_2_(SIRI) and the risk of OA. Furthermore, to investigate the threshold effect of log_2_(SIRI) on the risk of OA and to find the inflection point, smoothing curve fitting and generalized additive models were used.

Finally, further stratified and interaction analyses were conducted for age, sex, smoke status, alcohol consumption, hypertension, DM, and WHtR. All statistical tests were two-sided, with *p* < 0.05 indicating statistical significance.

## 3 Results

### 3.1 Basic characteristics of participants

This study enrolled 11,381 participants aged ≥20 years, including 1,437 (14%) with OA. Significant statistical differences were observed in baseline characteristics across quartiles of SIRI regarding age, sex, race, education, smoking, drinking, BMI, WHtR, hypertension, and DM (*p* all <0.001), as shown in Table 1.

**Table 1:** The baseline characteristics of the participants included in the study (*N* = 11,381).

		Quartiles of log_2_ (SIRI)				
Characteristic	Overall	Q1	Q2	Q3	Q4	*p* value
	*N* = 11,381	*N* = 3,191	*N* = 2,816	*N* = 2,618	*N* = 2,756	
Age (years)	45(20–85)	42(20–85)	43(20–85)	46(20–85)	49(20–85)	<0.001
Sex						<0.001
Female	5,503 (49%)	1,781 (57%)	1,401 (50%)	1,192 (46%)	1,129 (42%)	
Male	5,878 (51%)	1,410 (43%)	1,415 (50%)	1,426 (54%)	1,627 (58%)	
Age (years)						<0.001
20–59	8,122(77%)	2,488 (83%)	2,098 (81%)	1,843 (76%)	1,693 (68%)	
≥60	3,259 (23%)	703 (17%)	718 (19%)	775 (24%)	1,063 (32%)	
Race						<0.001
Non-Hispanic White	4,904 (68%)	919 (56%)	1,168 (68%)	1,274 (73%)	1,543 (76%)	
Non-Hispanic Black	2,194 (9.9%)	968 (18%)	497 (9.0%)	372 (6.5%)	357 (6.3%)	
Mexican American	1,900 (9.1%)	530 (10%)	531 (10%)	447 (8.5%)	392 (7.6%)	
Other/multiracial	1,207 (6.9%)	468 (9.8%)	301 (7.0%)	234 (5.9%)	204 (4.9%)	
Other Hispanic	1,176 (5.7%)	306 (5.9%)	319 (6.1%)	291 (5.7%)	260 (5.2%)	
Education						<0.001
Above High School	6,222 (63%)	1,825 (66%)	1,541 (64%)	1,439 (64%)	1,417 (57%)	
Below High School	2,613 (15%)	689 (14%)	662 (14%)	608 (14%)	654 (17%)	
High School	2,546 (23%)	677 (20%)	613 (22%)	571 (22%)	685 (26%)	
Smoke						<0.001
Current smoker	2,299 (19%)	462 (13%)	517 (17%)	553 (20%)	767 (27%)	
Former smoker	2,657 (25%)	626 (21%)	642 (24%)	628 (25%)	761 (28%)	
Never smoker	6,425 (56%)	2,103 (66%)	1,657 (59%)	1,437 (55%)	1,228 (45%)	
Drink						<0.001
<12 drinks/year	10,179 (91%)	2,937 (93%)	2,530 (92%)	2,325 (91%)	2,387 (89%)	
≥12 drinks/year	1,202 (8.8%)	254 (6.6%)	286 (7.7%)	293 (9.4%)	369 (11%)	
BMI	29 ± 6	28 ± 6	28 ± 6	29 ± 7	30 ± 7	<0.001
BMI						<0.001
Normal	3,402 (31%)	1,109 (38%)	845 (32%)	708 (27%)	740 (26%)	
Overweight	3,937 (34%)	1,114 (34%)	974 (34%)	899 (34%)	950 (34%)	
Obese	4,042 (35%)	968 (28%)	997 (34%)	1,011 (39%)	1,066 (40%)	
Hypertension						<0.001
Yes	3,616 (29%)	835 (23%)	791 (26%)	862 (30%)	1,128 (39%)	
None	7,765 (71%)	2,356 (77%)	2,025 (74%)	1,756 (70%)	1,628 (61%)	
DM						<0.001
Yes	1,241 (8.2%)	268 (5.9%)	271 (6.0%)	297 (8.4%)	405 (12%)	
None	10,140 (92%)	2,923 (94%)	2,545 (94%)	2,321 (92%)	2,351 (88%)	
WHtR	0.58 ± 0.09	0.56 ± 0.09	0.57 ± 0.09	0.59 ± 0.09	0.60 ± 0.10	<0.001
WHtR						<0.001
Exceeds Standard	9,378 (80%)	2,452 (74%)	2,307 (78%)	2,241 (85%)	2,378 (85%)	
Normal	2,003 (20%)	739 (26%)	509 (22%)	377 (15%)	378 (15%)	
SIRI	1.16 ± 0.79	0.50 ± 0.13	0.82 ± 0.08	1.16 ± 0.12	2.16 ± 0.95	<0.001
log_2_ (SIRI)	1.04 ± 0.42	0.58 ± 0.13	0.86 ± 0.07	1.11 ± 0.08	1.61 ± 0.34	<0.001
OA						<0.001
None	9,944 (86%)	2,886 (90%)	2,503 (89%)	2,255 (85%)	2,300 (83%)	
Yes	1,437 (14%)	305 (10%)	313 (11%)	363 (15%)	456 (17%)	

Continuous variables are described as means and SD, and categorical variables are presented as numbers (percentages).BMI, Body mass index; DM, Diabetes mellitus; WHtR, Waist-to-Height Ratio; SIRI, Systemic inflammation response index; OA, Osteoarthritis.A *p* value less than 0.05 indicates that there is a significant difference in the variable among at least one of the groups compared to the others. Age are presented as median (Min-Max range).

According to Supplementary Table S1, the following characteristics can be summarized: non-Hispanic White was the most common race; OA prevalence (%) increased with participants’ age; OA (%) was significantly more prevalent in female participants than in males. The prevalence of OA (%) among participants with different smoking, drinking, hypertension, DM, and visceral adiposity index statuses also showed significant differences (*p* < 0.001).

### 3.2 Univariate logistic regression analysis for OA

**Figure 3:**
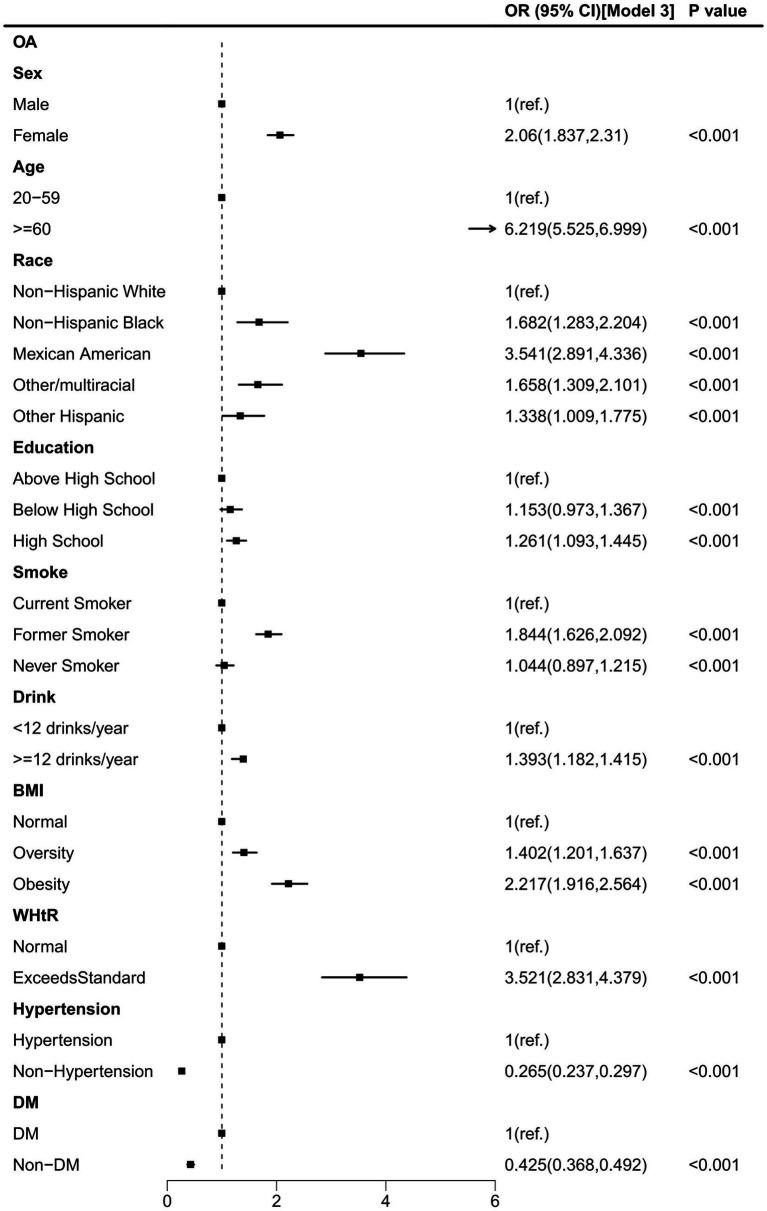
Weighted univariate logistic analysis of OA.

Figure 3 presents the results of the univariate logistic regression analysis for OA. Our findings showed that individuals aged ≥60 years, females, Mexican Americans, those with a high school education level, former smokers, those consuming ≥12 drinks/year, and those classified as obese (BMI >30 kg/m^2^) or exceeding the standard WHtR (WHtR > 0.5), had a significantly increased risk of OA (all OR > 1, all *p* < 0.001). Conversely, participants categorized as non-hypertensive and non-diabetic exhibited a significantly reduced risk of OA (all OR < 1, all *p* > 0.05).

### 3.3 Analysis of the relationship between OA and SIRI

**Figure 4:**
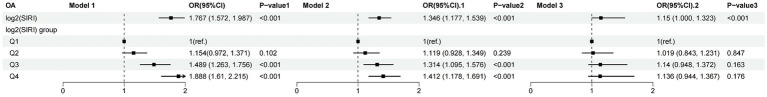
Relationship between OA and SIRI. Model 1: unadjusted. Model 2: adjusted for age, gender, and race. Model 3: based on Model 2, further adjusted for educational, smoke, drinker, hypertension, diabetes, BMI, and WHtR.

Figure 4 shows the results from the weighted multivariable regression analysis, indicating a significant correlation between higher levels of SIRI and an increased risk of OA (*p* < 0.001). This association was evident in the unadjusted Model 1 (OR: 1.706; 95% CI: 1.572–1.987, *p* < 0.001) and Model 2, which was adjusted for age, sex, and race (OR: 1.346; 95% CI: 1.117–1.539, *p* < 0.001). In the fully adjusted Model 3, log_2_(SIRI) continues to be positively correlated with OA (OR: 1.150; 95% CI: 1.000–1.323, *p* < 0.05), suggesting that each unit increase in log_2_(SIRI) was associated with a 15% increase in the risk of OA.

Further sensitivity analysis was conducted by categorizing log_2_(SIRI) from a continuous to a categorical variable (quartiles), as shown in Figure 4. The results in the unadjusted Model 1 revealed that compared to the lowest quartile (Q1), the highest quartile (Q4) saw an 88.8% increase in OA risk (OR: 1.888; 95% CI: 1.610–2.215, *p* < 0.001). In Model 2, adjusted for age, sex, and race, the risk of OA was 41.2% higher in the highest quartile (Q4) compared to the lowest quartile (Q1) (OR: 1.412; 95% CI: 1.178–1.691, *p* < 0.001). The relationship between OA and log_2_(SII) is shown in Supplementary Table S2; a significant correlation was only found in the unadjusted model (OR: 1.400; 95% CI: 1.200–1.634, *p* < 0.001).

### 3.4 Nonlinear relationship between OA and SIRI

**Figure 5:**
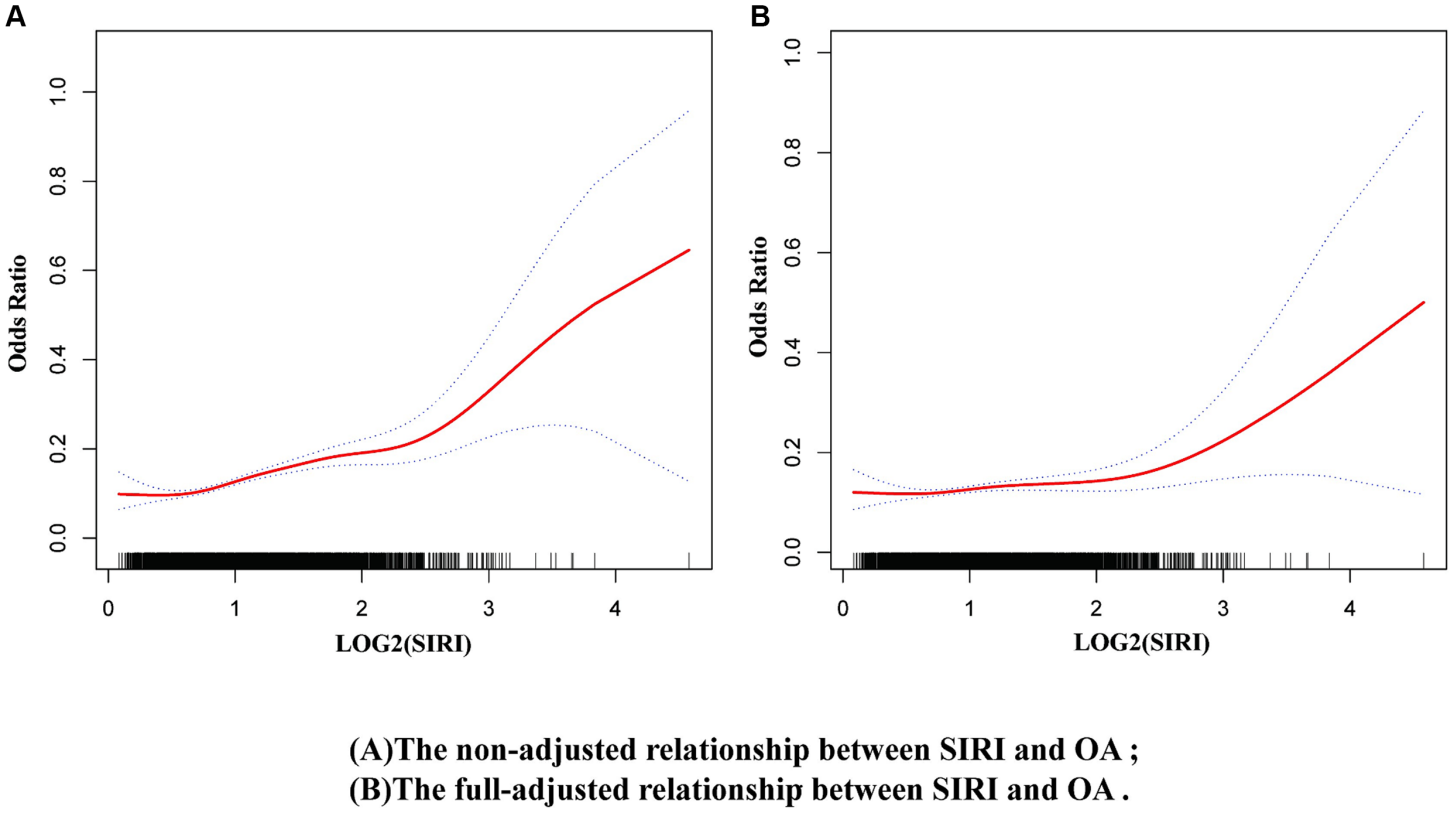
Nonlinear relationship between OA and SIRI. **(A)** Non-adjusted model, **(B)** Full-adjusted model.

Using the restricted cubic spline model in the original model, a nonlinear relationship was observed between SIRI and OA risk (Figure 5A). This nonlinear relationship persisted even after full adjustment (Figure 5B). Additionally, a threshold effect was observed (Supplementary Table S3), with the inflection point for log2(SIRI) = 2.25 (SIRI = 4.757). When log_2_(SIRI) was <2.25 (i.e., SIRI < 4.757), the risk of OA remained nearly unchanged; however when log_2_(SIRI) was >2.25 (i.e., SIRI > 4.757), the risk of OA increased rapidly. Supplementary Figure S1 shows the nonlinear relationship between OA and log_2_(SII).

### 3.5 Subgroup analysis and interaction tests

**Figure 6:**
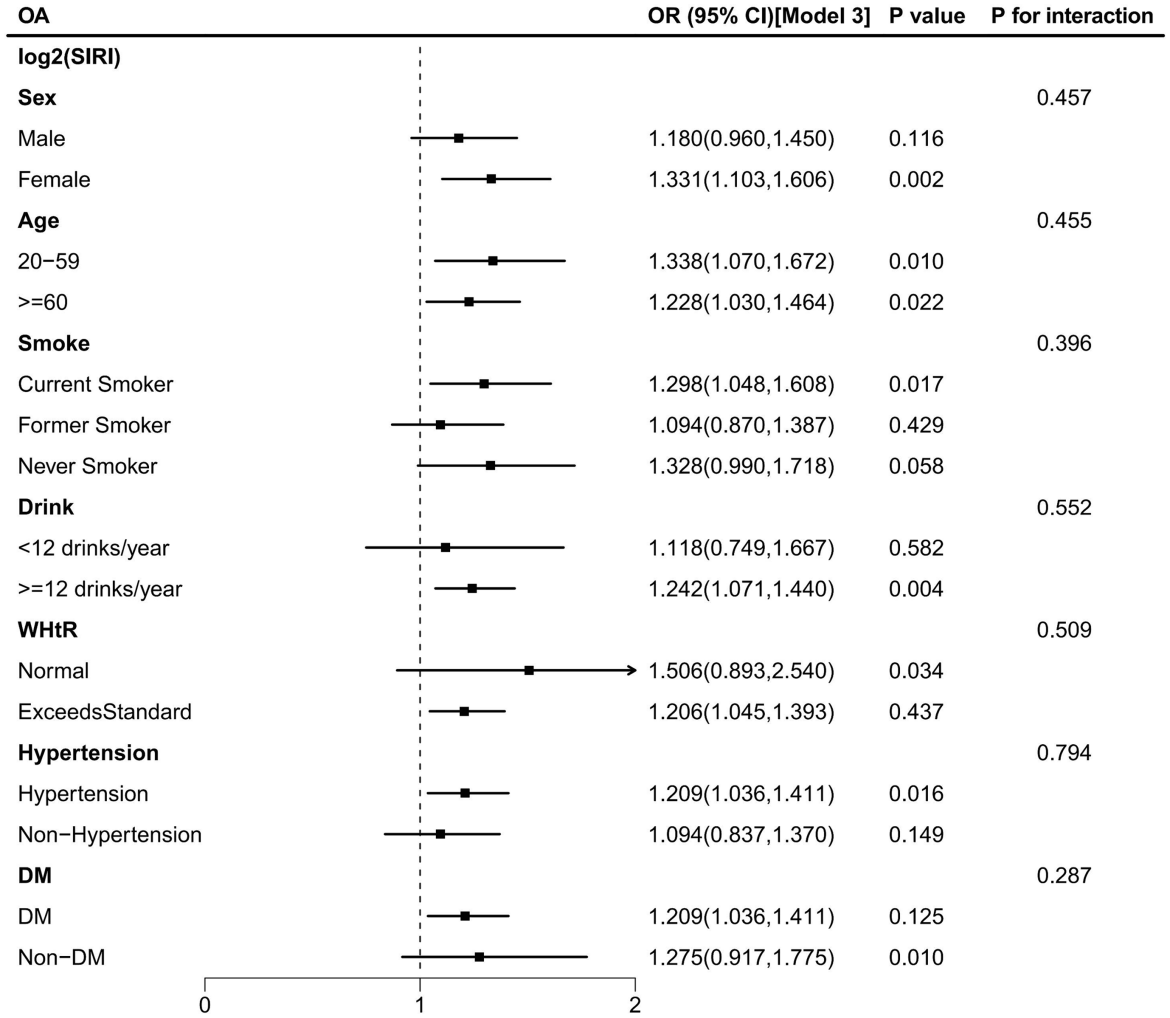
Subgroup analysis and interaction test for the association between SIRI and OA.

The study found that the association between elevated levels of SIRI and OA risk was not consistent across subgroups (Figure 6). For males, former smokers, never smokers, <12 drinks/year drinkers, non-hypertensive, non-DM, and normal WHtR (WHtR < 0.5) participants, the correlation was not statistically significant (*p* > 0.05). Furthermore, interaction tests revealed that sex, age, smoking status, drinking status, WHtR (obesity), hypertension, and DM did not significantly affect this association (Figure 6, all *p* for interaction >0.05).

## 4 Discussion

This study included 11,381 participants from the NHANES database spanning 2005–2018, comprising 5,503 females and 5,878 males. Among the included participants, 1,437 (14%) were diagnosed with OA. Compared to the non-OA participants, those with OA had higher SIRI levels. Moreover, after adjusting for all covariates, the relationship exhibited nonlinearity. In addition, we found that when SIRI was below 4.757, the risk of OA remained nearly unchanged; however, when SIRI exceeded 4.757, the risk of OA increased rapidly. This effect was more pronounced among females, current smokers, those consuming ≥12 drinks/year, and participants with hypertension, DM, and a WHtR exceeding the standard (WHtR >0.5) (all *p* < 0.05). In addition, compared to SII, SIRI showed a stronger correlation with OA.

To date, this study is the first to analyze the correlation between SIRI levels and the risk of OA using the NHANES database. OA is a degenerative joint disease prevalent in individuals over 60 years, where the incidence and disability rates can reach as high as 50 and 53%, respectively (19). Our study confirms this, as the incidence of OA among participants over 60 years was 57% (940 out of 1,437 cases). Key pathological mechanisms of OA include cartilage degeneration, bone proliferation, osteophyte formation, joint space narrowing, and degenerative inflammation (20). Factors such as sex, age, obesity, and inflammation are currently believed to be closely related to OA (21). Our study also supports these views, showing a significantly higher proportion of females with OA at 64% (917 out of 1,437 cases) compared to males and participants with obesity at 93% (1,346 out of 1,437 cases) compared to their non-obese counterparts.

Immunoinflammatory processes play a crucial role in the onset and progression of OA, making the control of inflammatory responses significant for the prevention and treatment of OA. Inflammatory cytokines disrupt the balance between catabolic and anabolic processes in joint tissues, leading to progressive degradation of joint cartilage, which is critical for biomechanical functions and ultimately results in the gradual loss of joint function and pain (22, 23). Interleukin (IL)-1β, tumor necrosis factor (TNF)-α, and IL-6, primarily produced by synovial and cartilage cells, inhibit the synthesis of collagen and proteoglycans in chondrocytes. They also promote the production of matrix metalloproteinases (MMPs), aggrecan, A disintegrin and metalloproteinase with thrombospondin motifs, and key inflammatory and destructive mediators like cyclooxygenase-2, prostaglandin E2, and inducible nitric oxide synthase, leading to extracellular matrix degradation or reduced production and chondrocyte apoptosis. This process further contributes to cartilage damage and exacerbates symptoms in patients with OA (24–26). Similarly, IL-1β, TNF-α, and IL-6 induce the production of other cytokines, MMPs, and prostaglandins while inhibiting the synthesis of proteoglycan and type II collagen, playing a key role in cartilage matrix degradation and bone resorption in OA. Moreover, these cytokines may indirectly contribute to OA through the regulation of adipocyte-derived factors like adiponectin and leptin (27). C-reactive protein (CRP) and erythrocyte sedimentation rate (ESR) are commonly used laboratory markers of systemic inflammation, typically elevated in inflammatory joint diseases like rheumatoid arthritis. Historically, OA was considered a non-inflammatory joint disease, where inflammatory serum markers such as CRP were not elevated. However, recent studies have shown that high-sensitivity-CRP and ESR are elevated in patients with OA and are closely associated with the disease progression and prognosis (28, 29).

Recently, SIRI has emerged as a novel systemic inflammation biomarker whose prognostic value for disease outcomes has been demonstrated in various types of cancer (11–13). However, no studies have yet reported the relationship between SIRI and OA. SIRI was found to exhibit a right-skewed, non-normal distribution, leading to the application of logarithmic transformation before data analysis. The results indicate that SIRI measures the level of systemic inflammation in patients with OA and has proven to be a strong predictor of disease activity, joint damage, and radiographic progression. Our results suggest that higher SIRI levels are significantly associated with increased risk of OA, highlighting the potential for using SIRI as a biomarker for early detection of OA. This could enable healthcare providers to identify individuals at higher risk for developing OA and implement preventive measures sooner, potentially slowing disease progression. Regular screening of SIRI levels in at-risk populations, such as older adults or those with a family history of OA, could be integrated into routine clinical practice. Monitoring SIRI levels over time might also help in assessing the effectiveness of preventive interventions. Given the strong link between inflammation and OA, the study supports the need for anti-inflammatory therapies as part of the treatment regimen for OA patients. Therapies targeting systemic inflammation could be beneficial in managing OA symptoms and improving patient outcomes.

The strength of this study lies in its being the first large-scale study based on the NHANES database to explore the relationship between SIRI and OA. Considering that the NHANES database comprises multi-stage complex sampling data, weighted logistic regression models were used in the analysis, and all other covariates were adjusted. Additionally, SIRI was log_2_-transformed before analysis to ensure a normal distribution. Furthermore, we conducted a comprehensive subgroup analysis and explored the potential influencing factors according to gender, age, smoking, alcohol consumption, hypertension, and diabetes. Finally, to explore the nonlinear relationship between SIRI and OA, restricted cubic spline models, smoothing curve fitting, and generalized additive models were employed.

This study has some limitations. First, some variables in the study were obtained through surveys and self-reporting, which can introduce bias. Second, the study uses a cross-sectional design, which limits the ability to establish causality between SIRI levels and OA risk. Third, there may be unmeasured confounders that could influence both SIRI levels and OA, such as dietary habits, physical activity levels, or genetic factors. Fourth, the study population is based on NHANES data, which is representative of the United States population. The findings may not be generalizable to populations with different demographics or healthcare systems. Moreover, the NHANES database does not include classic inflammatory markers such as IL-6, TNF-α, and IL-10, limiting the ability to conduct a more comprehensive analysis with additional indicators. Therefore, it is hoped that this study will provide data support for future scientific research and encourage more researchers and clinicians to explore inflammatory biomarkers in OA.

## 5 Conclusion

This study provides new insights into the relationship between SIRI and OA. A positive correlation existed between SIRI levels and the risk of OA, with a critical value of 4.757, which underscores the importance of timely anti-inflammatory treatment when the SIRI value exceeds 4.757 to prevent the onset of OA. Furthermore, our research demonstrates that SIRI is a novel, clinically valuable, and convenient inflammatory marker that can predict the risk of OA in adults. In addition to these attributes, SIRI offers the advantages of being low-cost, easy to collect, and simple to calculate. Overall, we hope that SIRI can become a clinical assessment tool for predicting OA risk.

## Data availability statement

The original contributions presented in the study are included in the article/Supplementary material, further inquiries can be directed to the corresponding authors.

## Ethics statement

The studies involving human participants were reviewed and approved by The ethics review board of the National Center for Health Statistics. The patients/participants provided their written informed consent to participate in this study.

## Author contributions

QH: Conceptualization, Data curation, Formal analysis, Writing – original draft. ZW: Methodology, Validation, Writing – original draft. JM: Conceptualization, Writing – review & editing. CX: Software, Supervision, Writing – review & editing. XS: Funding acquisition, Project administration, Resources, Writing – review & editing.

## Footnotes

^1^ https://www.cdc.gov/nchs/nhanes/index.htm

## Supplementary material

Supplementary Figure S1 Nonlinear relationship between OA and SII. **(A)** Non-adjusted model, **(B)** Full-adjusted model.
