# Supplementary figures and images for: Relationship between systemic immune-inflammation index and osteoarthritis: a cross-sectional study from the NHANES 2005–2018
Source: Front Med (Lausanne). 2024 Aug 14;11:1433846. doi: 10.3389/fmed.2024.1433846 (PMC11349521; doi:10.3389/fmed.2024.1433846)

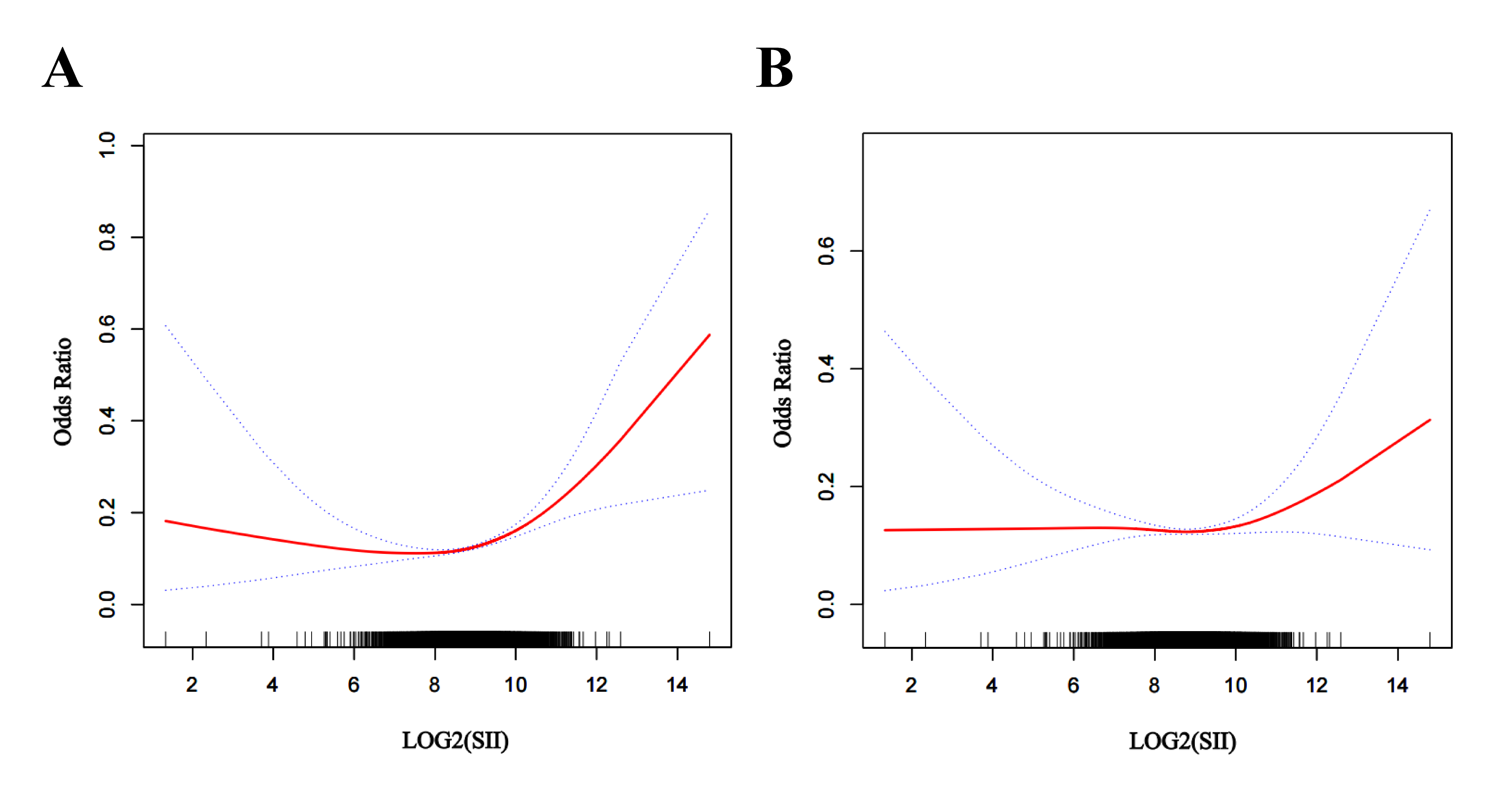

Supplement: Supplementary Figure S1 — Nonlinear relationship between OA and SII. (A) Non-adjusted model, (B) Full-adjusted model. [file Image_1.TIF]
