# Supplementary material for: Relationship between systemic immune-inflammation index and osteoarthritis: a cross-sectional study from the NHANES 2005–2018
Source: Front Med (Lausanne). 2024 Aug 14;11:1433846. doi: 10.3389/fmed.2024.1433846 (PMC11349521; doi:10.3389/fmed.2024.1433846)
Supplement: Supplementary file 2 [file Table_1.DOCX]

**Table S1**.Basic characteristics of participants

| Characteristic | Overall,  N = 11381 (100%) | Non-Osteoarthritis  N = 9944 (86%) | Osteoarthritis  N = 1437 (14%) | *p-*value |
| --- | --- | --- | --- | --- |
| Age (years) | 45(20-85) | 42(20-85) | 62(22-85) | <0.001 |
| Sex |  |  |  | <0.001 |
| Female | 5,503 (49%) | 4,586 (46%) | 917 (64%) |  |
| Male | 5,878 (51%) | 5,358 (54%) | 520 (36%) |  |
| Age (years) |  |  |  | <0.001 |
| 20-59 | 8,122 (77%) | 7,625 (82%) | 497 (43%) |  |
| ≥60 | 3,259 (23%) | 2,319 (18%) | 940 (57%) |  |
| Race |  |  |  | <0.001 |
| Non-Hispanic White | 4,904 (68%) | 4,000 (66%) | 904 (83%) |  |
| Non-Hispanic Black | 2,194 (9.9%) | 1,984 (11%) | 210 (5.8%) |  |
| Mexican American | 1,900 (9.1%) | 1,786 (10%) | 114 (3.1%) |  |
| Other/multiracial | 1,207 (6.9%) | 1,112 (7.2%) | 95 (5.2%) |  |
| Other Hispanic | 1,176 (5.7%) | 1,062 (6.2%) | 114 (2.8%) |  |
| Education |  |  |  | 0.2 |
| Above High School | 6,222 (63%) | 5,387 (62%) | 835 (65%) |  |
| Below High School | 2,613 (15%) | 2,327 (15%) | 286 (13%) |  |
| High School | 2,546 (23%) | 2,230 (23%) | 316 (22%) |  |
| Smoke |  |  |  | <0.001 |
| Current smoker | 2,299 (19%) | 2,041 (20%) | 258 (18%) |  |
| Former smoker | 2,657 (25%) | 2,172 (23%) | 485 (35%) |  |
| Never smoker | 6,425 (56%) | 5,731 (57%) | 694 (48%) |  |
| Drink |  |  |  | <0.001 |
| <12 drinks/year | 10,179 (91%) | 8,937 (92%) | 1,242 (88%) |  |
| ≥12 drinks/year | 1,202 (8.8%) | 1,007 (8.3%) | 195 (12%) |  |
| BMI |  |  |  | <0.001 |
| Normal | 3,402 (31%) | 3,112 (32%) | 290 (22%) |  |
| Overweight | 3,937 (34%) | 3,482 (35%) | 455 (31%) |  |
| Obese | 4,042 (35%) | 3,350 (33%) | 692 (47%) |  |
| Hypertension |  |  |  | <0.001 |
| Hypertension | 3,616 (29%) | 2,765 (25%) | 851 (55%) |  |
| Non-Hypertension | 7,765 (71%) | 7,179 (75%) | 586 (45%) |  |
| DM |  |  |  | <0.001 |
| DM | 1,241 (8.2%) | 954 (7.0%) | 287 (15%) |  |
| Non-DM | 10,140 (92%) | 8,990 (93%) | 1,150 (85%) |  |
| WHtR | 0.58 ± 0.09 | 0.58 ± 0.09 | 0.63 ± 0.10 | <0.001 |
| WHtR |  |  |  | <0.001 |
| Exceeds Standard | 9,378 (80%) | 8,032 (78%) | 1,346 (93%) |  |
| Normal | 2,003 (20%) | 1,912 (22%) | 91 (7.1%) |  |
| SIRI | 1.16 ± 0.79 | 1.13 ± 0.75 | 1.34 ± 0.99 | <0.001 |
| log2_SIRI | 1.04 ± 0.42 | 1.02 ± 0.41 | 1.14 ± 0.47 | <0.001 |
| BMI: body mass index; DM: Diabetes mellitus; WHtR:Waist-to-Height Ratio; SIRI: Systemic Inflammation Response Index. Age are presented as median (Min-Max range) | | | | |

|  | ***β (95% CI) p-*value** | | |
| --- | --- | --- | --- |
|  | Model 1 | Model 2 | Model 3 |
| Osteoarthritis (OA) |  |  |  |
| log2(SII) | 1.207(1.123, 1.297)  <0.001 | 1.083 (1.004, 1.168)  0.039 | 0.999(0.925, 1.097)  0.974 |
| Waist-to-Height Ratio (WHtR) quartiles | | | |
| Q1 | 1(ref.) | 1(ref.) | 1(ref.) |
| Q2 | 0.973 (0.825, 1.146)  0.741 | 0.932 (0.781, 1.113)  0.435 | 0.877 (0.732, 1.052)  0.157 |
| Q3 | 1.138 (0.970, 1.335)  0.114 | 1.022 (0.860, 1.216)  0.802 | 0.915 (0.766, 1.093)  0.326 |
| Q4 | **1.400 (1.200, 1.634)**  **<0.001** | 1.134 (0.958, 1.342)  0.144 | 0.954 (0.802, 1.135)  0.595 |
| ***p for trend*** | <0.001 | 0.070 | 0.798 |
| Model 1: Unadjusted.  Model 2: Adjusted for Age, Gender, and Race.  Model 3: Based on Model 2, further adjusted for Educational, Smoke, Drink, Hypertension, Diabetes, BMI, and WHtR. | | | |

**Table S2.** Relationship between OA and SII.

| Saturation effect analysis | **Odds ratio *(95% CI) p-*value** |
| --- | --- |
| K value |  |
| log2(SIRI) | **2.25** |
| SIRI | **4.757** |
| log2(SIRI) <K, effect 1 | 1.148 (0.987, 1.336) *p*=0.074 |
| log2(SIRI) >K, effect 2 | 2.670 (1.230, 5.798) ***p*=0.013** |
| ***p* for Log-likelihood ratio** | **0.048** |

**Table S3**.The threshold effect of the nonlinear relationship between OA and SIRI
